# From waste to resource: A systems dynamics and stakeholder analysis of phosphorus recycling from municipal wastewater in Europe

**DOI:** 10.1007/s13280-018-1097-9

**Published:** 2018-09-14

**Authors:** Claudiu-Eduard Nedelciu, Kristín Vala Ragnarsdóttir, Ingrid Stjernquist

**Affiliations:** 1 Institute of Earth Sciences, University of Iceland, Askja, Sturlugata 7, Reykjavík, 101 Iceland; 2 Department of Physical Geography, Stockholm University, 106 91 Stockholm, Sweden

**Keywords:** Case study, Phosphorus, Recycling, System dynamics, Stakeholder analysis, Wastewater

## Abstract

Recycling Phosphorus (P) from urban wastewater can secure part of domestic agricultural P supply and contribute to a circular P supply chain. In this paper, we use literature review, stakeholder interviews and analysis, and systems dynamics for the capital cities of Stockholm and Budapest as case studies. We find that political support is a prerequisite for developing the P recycling sector, and policy makers are the most influential stakeholders. P criticality is the main driver for political support. P externalities from mining to sludge disposal should be considered when evaluating P criticality and recycling profitability. We conclude with policy recommendations for the development of the P recycling sector, arguing for legally binding P recycling targets and prioritization of the safest technological solutions available. Our analysis identifies three policy action indicators and five policy interventions in the recycling system.

**Electronic supplementary material:**

The online version of this article (10.1007/s13280-018-1097-9) contains supplementary material, which is available to authorized users.

## Introduction

Phosphorus (P) is an essential macronutrient needed for plant growth. In agriculture, more than 85% of the P fertilizer comes from mined phosphate rock (PR) (Cordell et al. 2009). PR is mined from a very limited number of countries, most notably Morocco, China, and the United States of America (USA). According to the latest report of the United States Geological Survey (USGS), close to 74% of the world’s reserves of PR are found in Morocco and Western Sahara (USGS 2018). The European Union’s import dependency on PR was estimated at 92% in 2011 (EU Commission 2013), prompting the European Commission to include P in the list of Critical Raw Materials (CRMs) in 2014. This means that PR is now considered a high supply-risk and high economic value raw mineral. P fertilizer prices were also a determining factor for this decision. In 2007–2008 a 400% increase in P fertilizer prices sent a shockwave to the world market and attracted increased attention from the media, scientific community, and policy makers (Cordell et al. 2009; The Guardian 2010; Cordell and White 2014). The main factors for the price spike are many and include decreased P fertilizer production in the US; an increased export tax on P fertilizer, especially from China; increased oil and energy prices; disproportionate fertilizer demand for biofuel production; and disproportionate supply–demand relation (Scholz et al. 2014).

There are also differences in the heavy metal concentration—in particular cadmium (Cd)—between different deposits of PR. Purity of sedimentary PR, which accounts for almost 95% of the world resources, is much lower than that of magmatic deposits. The former usually exceed 60 mg Cd kg^−1^ PR and the latter are around or less than 10 mg Cd kg^−1^ PR (GTK 2017). Concentrations of Cd in soil depend on Cd deposition as well as Cd concentration of fertilizers and their application rates. Increasing Cd concentrations in soil have been shown to lead to increased Cd concentration in crops (Roberts 2014). Cd in food can have an adverse effect on human health, especially in the form of kidney disease, but harmful effects on the musculoskeletal system are also documented (Roberts 2014). A 2013 report of the Swedish Chemicals Agency estimates the economic cost of bone fractures caused by dietary Cd exposure in Sweden at 4.2 billion SEK (app. 420 million euros) a year (KEMI 2013). At present, Europe is importing most of its PR from Morocco and Algeria, both of which have sedimentary reserves (EU Commission 2013).

**Fig. 1:**
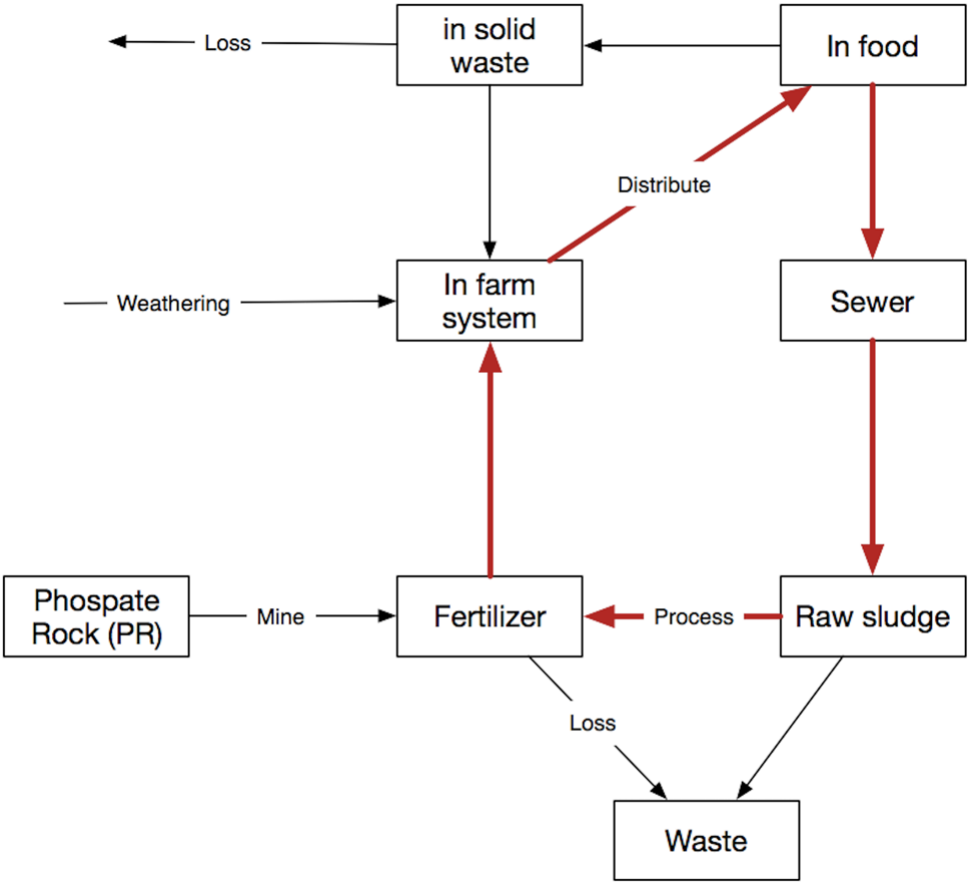
Flowchart of the phosphorus (P) supply chain. Boxes refer to P stocks, while arrows refer to P flows. P recycling from urban wastewater is in red

P is following a linear path from mining sites to wastewater effluent or disposal as some form of solid waste (Fig. 1). This means that the P input in the agricultural/food system is to a large extent not recovered and it causes a considerable harm to the environment. P is one of the main causes of eutrophication and the creation of “dead zones” in coastal areas (Chowdhury et al. 2017). Thus, P starts its life cycle as a natural resource retrieved at great environmental costs and ends as pollutant.

One solution to solve the P issue is to transform the P cycle into a circular rather than linear process (Ragnarsdottir et al. 2011). A leverage point identified in the literature is the wastewater (WW) stream (Mihelcic et al. 2011; Cordell and White 2014) from which P can be recovered and then recycled. In Fig. 1, P recycling from urban wastewater is shown in red arrows: from sewer, P is recovered from raw sludge and processed into fertilizer. It is then used in farm systems for food production. Part of the food ultimately reaches the sewers through human waste disposal and the process starts again. Studies show that up to 20% of the European P demand could be supplied by recycling P from municipal wastewater (EU Commission 2017a). Countries like Germany and Switzerland have recently introduced legally binding targets to recycle P from wastewater (EU Commission 2016; Swiss Federal Council 2015). Following Circular Economy Package (CEP) principles, the new EU Fertilizer Regulation Revision also aims to boost large-scale production of fertilizer from domestic organic or secondary raw materials. At the same time, it is expected to impose lower EU-wide limits on heavy metal concentration in fertilizers (EU Commission 2017b).

The aim of this study is (1) to analyze the dynamics of P recycling from urban wastewater, using two European capitals as case studies, and (2) to provide policy recommendations at the national level that can contribute to the development of wastewater P recycling sector.

## Theoretical framework

Case studies allow the testing of hypotheses, while being an appropriate method in understanding dynamics in multidisciplinary sciences (Flyvbjerg 2011). We gathered data from 23 stakeholders, using semi-structured interviews. In deciding on the interviewee sample, we adopted the method proposed by Malterud and Guassora (2016), using the concept of “information power” to guide our sample size and representation. Because of the very specific nature and restricted size of the P recycling sector we focused on the quality and relevance of information rather than on the quantity of interviewees. We used stakeholder analysis (Brugha and Varvasovszky 2000) based on an influence-interest matrix of stakeholders in the decision-making process as perceived in the literature. The final version of this matrix (Fig. 5) emerged after analyzing interview data. Stakeholder sample and size were completed using a combination of insider information provided by interviewees and by applying targeted snowball sampling method (Heckathorn and Cameron 2017). The difference in sample composition between Budapest and Stockholm is due to the different stages of development of P recycling in the two locations. For example, in Budapest there was no sludge spreading or P recycling from municipal wastewater. Moreover, there was no involvement or interest on behalf of farmer associations or the food industry. Therefore, these stakeholders are not represented. We examined P recycling using systems thinking and systems dynamics. Systems dynamics is a method “of dealing with questions about the dynamic tendencies of a complex system, that is, the behavioral patterns they generate over time” (Meadows 1970).

## Materials and methods

### Study area

We chose Stockholm and Budapest as case studies for three reasons: (1) they reflect to a satisfying extent the differences in how the wastewater (WW) sector is approached across European Member States in the North and West on one hand and Central Eastern Europe (CEE), respectively; (2) both capitals are comparable in size: 1.5 million people in the urban area of Stockholm and 1.8 million people in the urban area of Budapest; and (3) they are places where a relevant sample of stakeholders was approachable by us as researchers.

#### Stockholm, Sweden

Based on the P flow dynamics in Sweden (Linderholm and Mattsson 2013) and the total P discharge in municipal wastewater (SCB 2018), P from sewage sludge could secure 20–22% of the total P needed for food production in the country. The Swedish Environmental Protection Agency (SEPA) has proposed a milestone target to the Swedish Government to recycle 40% of the P and 10% of the N from sewage onto agricultural land, by 2018. By 2016, 34% of the P from wastewater was recycled on farmland through sludge spreading (SCB 2018). The Swedish Water and Wastewater Association, The Federation of Swedish Farmers (LRF, also referred in this study as “farmer association”), The Swedish Food Federation, the Swedish Food Retailers Federation and SEPA as co-opted member developed a certification system for wastewater treatment plants (WWTPs) referred to as REVAQ. REVAQ aims to reduce the flow of dangerous substances reaching WWTPs, in order to provide for a sludge quality that is acceptable for agricultural use and thereby to obtain acceptance for spreading sludge on arable land. Digested sludge at REVAQ certified WWTPs needs to have concentrations of certain contaminants such as Cd at levels deemed safe. Cd in fertilizers is of particular importance in Sweden, where the limit of 44 mg Cd kg^−1^ P_2_O_5_ and 100 mg Cd kg^−1^ P is stricter than in the EU due to environmental and human health concerns (Roberts 2014). Sweden made considerable efforts in the past decades to tackle high heavy metal loads in sludge; concentrations for cadmium, silver, copper, zinc, mercury, and lead decreased by up to 90% since 1970s (Kirchmann et al. 2017). Currently, SEPA’s position is that recycling P has to be made by imposing much stricter concentration levels for certain heavy metals and organic contaminants, in order to avoid adverse effects on ecosystems and human health (Naturvårdsverket 2013).

#### Budapest, Hungary

In Hungary, the focus in Budapest and other major cities across the country is to remove the pollutants from urban wastewater and ensure that the effluent released in water bodies is in accordance with nationally agreed targets. The sludge produced in the process is removed and managed by contracted companies. There is no certification system for using sludge in agriculture. Forty-two percent of the sludge produced in the country is used in landscaping projects, a process referred to as “recultivation” but which has no links to agricultural production. A quarter of the sludge is mixed with other compost and used in landscaping, while approximately 17% is disposed on fields, which are not used for agricultural production (Garai, pers. comm. 2017). At present, there are no municipal or national targets to recycle P from sludge. There is no legislation or guidelines with regard to pharmaceuticals and other chemicals. The closest initiatives to P recycling are limited to pilot projects of nutrient capture in biomass in the form of plant and tree greenhouses in South Pest WWTP (Organica 2018) or algae at in North Pest WWTP (MAB 2018).

### Research process

We used qualitative research methods and systems thinking to shape our research process in four stages. First, we gathered data through literature review and 23 semi-structured interviews. Second, we used systems dynamics to analyze the data. We chose to illustrate our results using causal loop diagrams (CLD), in which each causal link has a polarity—this is the direction of effect that the influencing variable has on the influenced variable. The polarity of each feedback loop is essential in understanding system’s behavior. The perturbation of a loop may result in the magnification of the original effect (a reinforcing loop, R) or into an equilibrating response (a balancing loop, B). Third, we made a normative stakeholder analysis for the P recycling sector, using the influence-power matrix design as described by Reed et al. (2009). Fourth, we identify policy action indicators and policy interventions in our “Discussion” section.

The final stakeholder sample reflects key sectors impacting P recycling at the two locations (see Table 1). The guiding questions addressed during the semi-structured interviews revolved around four main topics: (1) P criticality; (2) Feasibility of P recycling; (3) Policy aspects of P recycling; and (4) Social and safety aspects of P recycling. The set of guiding questions can be found in Electronic Supplementary Material. Interviews lasted for an hour on average.

**Table 1:** Sample of stakeholders selected for semi-structured interviews. Numbers indicate the number of stakeholders interviewed, m stands for male and f for female. The interviews comprised 23 persons, 11 in Hungary, 12 in Sweden, 7 were women, 16 were men

Stakeholder	Stockholm	Budapest
Policy at national level	0	1 m
Policy at municipal level	1 f	0
WWTP administration	1 m, 1 f	3 m
Private sector	1 m	4 m
Academia	2 f, 3 m	1 f
Farmers association	2 m	0
Food industry	1 f	0
NGO	0	1 f, 1 m

Throughout the paper, we refer to P removal as P capture in sludge. We refer to P recovery as the process of extracting P from sludge for further use in any branch of the industry but agriculture and food production, and we refer to P recycling as the extraction of P from sludge and its return to farmland, or as the spreading of sludge on farmland. Also, in our analysis, discussion and conclusion “stakeholders” refer strictly to the interviewed stakeholders, unless otherwise specified.

## Results

### Systems synthesis of literature and interviews

#### P recycling on policy agenda

**Fig. 2:**
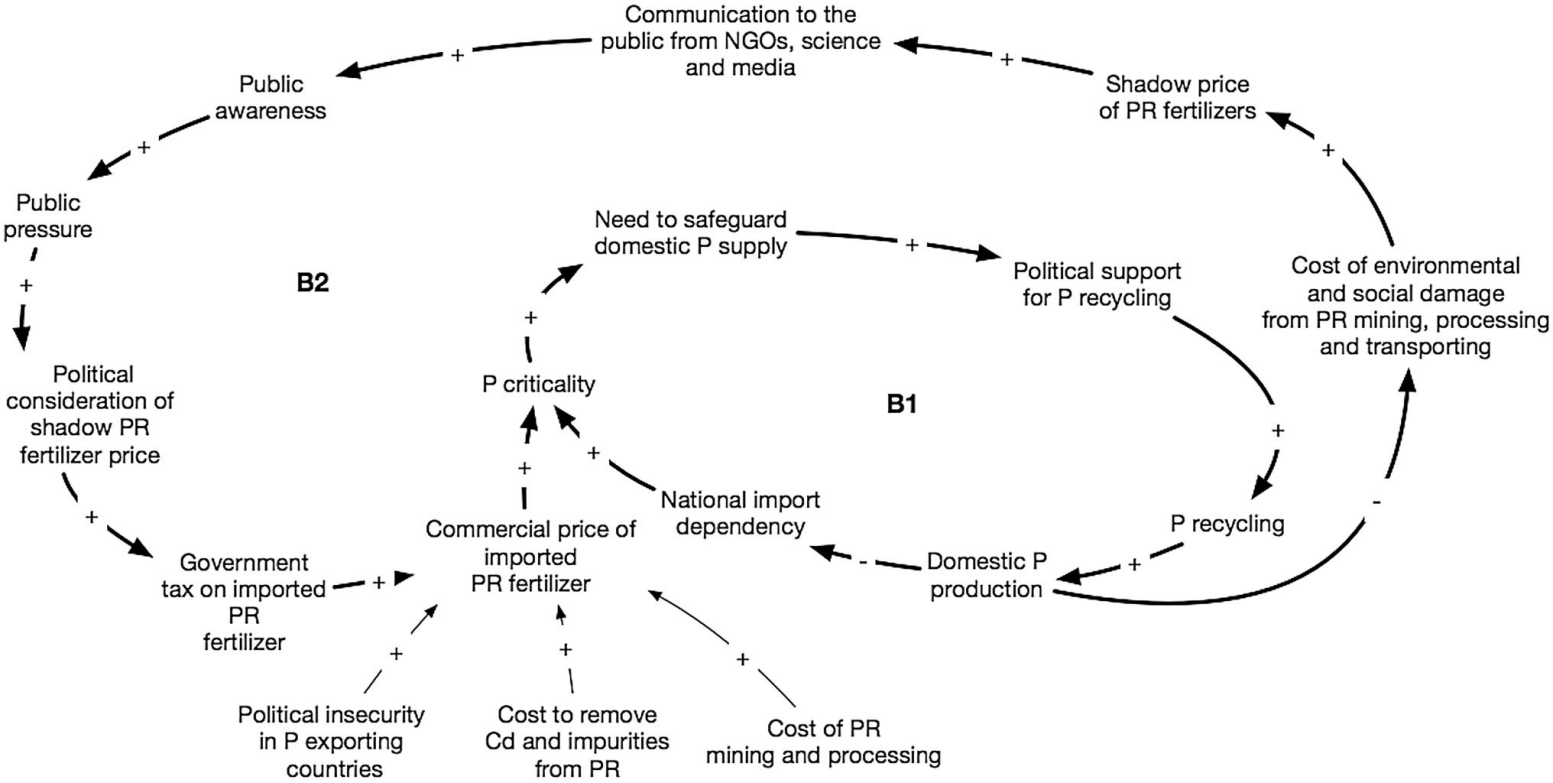
Causal loop diagram with two balancing loops (B1 and B2) showing how P recycling can get higher on policy agenda

All stakeholders in Budapest and Stockholm think that recycling P should be higher up on policy agendas. They believe that policy action is triggered by a high perceived P supply risk for the national agricultural sector. Throughout interviews “critical” or “criticality” are terms often mentioned. In the literature, criticality is seen as a matrix function of two axes: supply risk and vulnerability. Supply risk refers to the probability of disruption in the supply of a resource, while vulnerability is the impact of supply risk (Habib and Wenzel 2016). The European Commission also considers high supply risk, high environmental risk and high economic importance as key factors in identifying critical raw materials (EU Commission 2013). Thus, P criticality depends on a number of variables, but two are particularly significant: commercial price of PR fertilizer and national import dependency. These two variables start driving balancing loop B1 in Fig. 2.

Stakeholders identify three variables influencing commercial price of PR fertilizer (Fig. 2, non-bold arrows). First, they identify political insecurity in P exporting countries. Recurring examples given during interviews were the situation in PR-rich disputed territory of Western Sahara, the Arab Spring and the Syrian Civil War. In the literature, Cordell et al. (2015) and Allan (2016) also discuss the problematic of Western Sahara-Morocco conflict for the global P fertilizer supply. Second, they identify the cost of removing Cd and other heavy metals from PR. This particular aspect is important in the light of the new EU Fertilizer Regulation Revision (EU Commission 2017b). The Revision is likely to set ambitious low-level targets for Cd in phosphate fertilizers. Third, it is the cost of PR mining and processing. Specifically, stakeholders refer to costs for infrastructure development to increase production capacity and costs of other resources needed for PR processing. Scholz et al. (2014) also attributed the price spike in 2007–2008 partially to production capacity constraints and an increase in oil and energy prices. A fourth factor impacting commercial price of PR fertilizer derived from literature is government tax on PR fertilizer (Fig. 2, bold arrow). It is discussed as part of loop B2 below.

Stakeholders from the farmer association, national policy-making, private sector and academia believe that higher commercial price of PR fertilizer means higher food prices. They claim higher food prices are “political suicide” through loss of electorate if not civil unrest. In the literature, Bellemare (2015) finds that increase in food prices has led to increased social unrest across the world in the last few decades. All stakeholders in Stockholm and Budapest also argue that higher national import dependency makes farmers more vulnerable to global market price fluctuations, such as the ones in 2007–2008. They believe that higher national import dependency can jeopardize the delivery of optimal P supply for the domestic agricultural sector.

The balancing loop B1 in Fig. 2 continues with the rationale that high P criticality leads to awareness among lawmakers on the need to safeguard domestic P supply. This awareness materializes into political support for P recycling. For many of the stakeholders, political support translates into national binding targets for P recycling, similar to those already set in Germany and Switzerland. Such targets would increase the rate of domestic P recycling. Loop B1 is closed by an increase in domestic P production through higher P recycling rates. In this situation, P criticality decreases, which decreases the need for political support.

In Hungary, all stakeholders highlight the lack of a long-term vision on resource management by policy makers. In the words of a former lawmaker, the political establishment thinks, that “future generation problems need to be solved by future generations.” However, transition from political support for P recycling to optimal P recycling rates takes time. Interviewees estimate this interval to be one generation (20–25 years), while recent targets implemented in Germany and Switzerland indicate 10–15 years. Therefore, support for P recycling needs to be prepared and set in a timely manner.

Loop B2 in Fig. 2 is supported by the literature. Mining, processing and transport of PR require considerable amounts of resources such as water, Sulphur, energy, and materials to build new infrastructure. Mining of PR generates millions of tons of waste annually, including phosphate sludge, contributing to pollution of land and aquatic ecosystems (Cordell et al. 2015). There are also social costs to pay for PR mining, most notably community displacement and conflict. Thus, PR fertilizers are produced with a hidden cost of socio-environmental externalities (Cordell et al. 2015), which increases their shadow price and triggers loop B2. The higher the shadow price of PR fertilizers is, the more it fuels interest from NGOs, academia and media, who communicate it to the public. Increased public awareness leads to increased public pressure on lawmakers, who are thus likely to consider the shadow PR fertilizer prices. One way that governments can account for externalities is taxing. China imposed a tax on the export of PR in order to secure domestic supply (Scholz et al. 2014), thus reducing both import and export. Except in Finland, in Europe the tax could only apply to imported PR fertilizer. Such tax would increase the commercial price of PR fertilizer and in turn, increase P criticality (see Fig. 2). P criticality is where loop B2 merges with loop B1 and eventually leads to higher domestic P production through P recycling. A higher domestic P production will decrease international P externalities.

#### Developing P recycling from urban wastewater at a national level

**Fig. 3:**
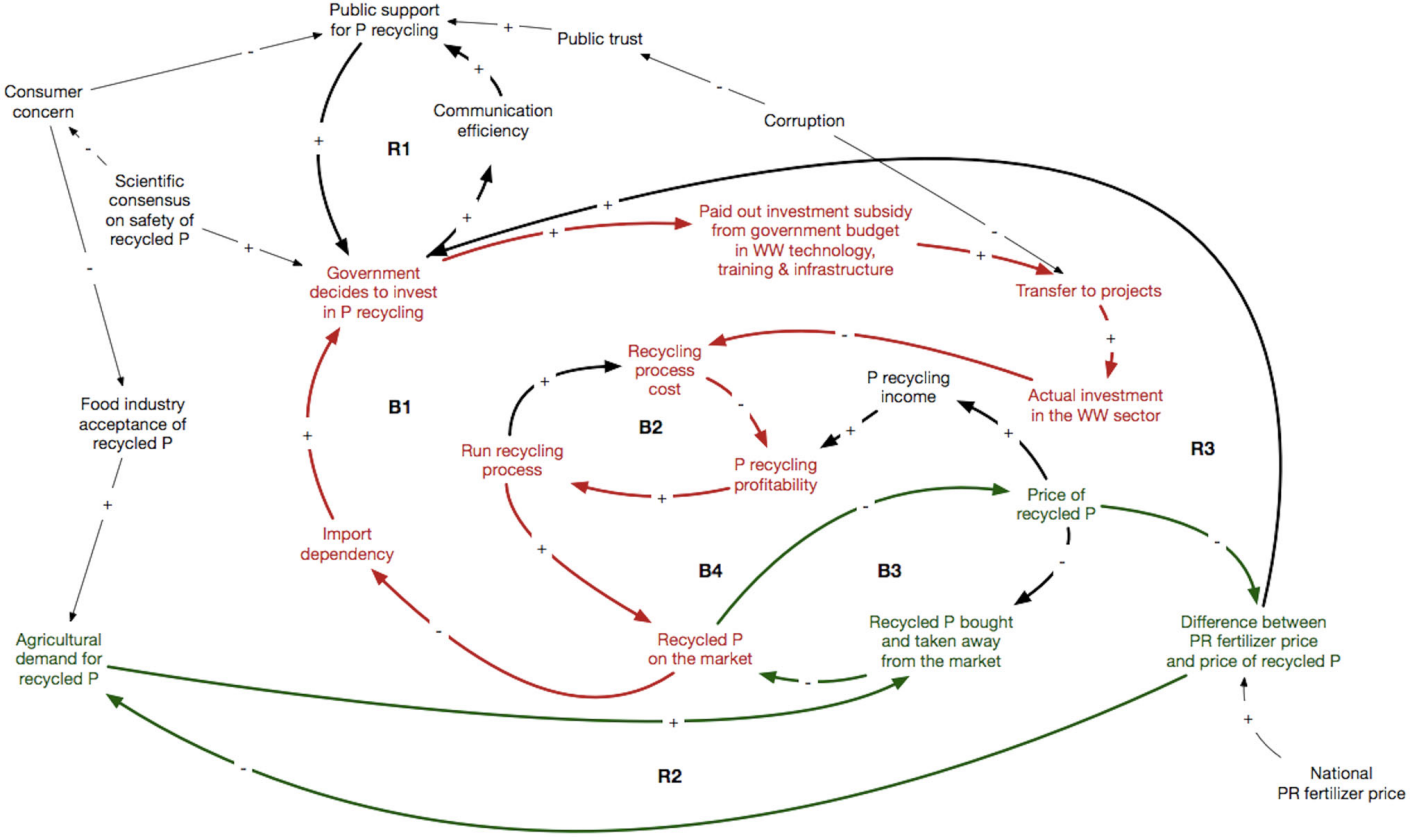
Causal loop diagram showing the main dynamics of P recycling implementation as identified from interviews and the literature. Red arrows are for the policy intervention in loop B1, green arrows are for the impact of the agriculture sector on recycled P market

Figure 3 shows the main dynamics for P recycling development at national level. Assumptions taken for this CLD are that (1) political support exists and there is sufficient available budget; and (2) WWTPs are operational. The first assumption stems from political support being a prerequisite of P recycling as shown in Fig. 2 and the understanding of stakeholders that state investment is essential to the overall development of the recycling sector. The second assumption stems from stakeholders’ assumptions.

All stakeholders believe political support should materialize in investment subsidies for wastewater infrastructure, wastewater technology and training of staff from the wastewater sector. These subsidies can also be directed at public–private partnerships or financing entrepreneurs in the wastewater sector. In loop B1 (in red), we show that this policy intervention results in decreased costs of the P recycling process and increased recycling profitability. It further enables an increased amount of recycled P to reach the market by intensifying the recycling process. More recycled P on the market decreases import dependency and the urgency of the government to decide on investing in the recycling sector, hence reducing investment subsidies.

However, the aim of policy intervention is twofold. Market mechanisms in loops B2, B3, and B4 lower the price of recycled P by increasing the amount of recycled P on the market. Loop R2 shows that as the difference between PR fertilizer prices and price of recycled P decreases, agricultural demand for recycled P increases, taking away recycled P from the market. This means that P recycling costs can be covered more by market revenues and less by government subsidies. The system then sends a feedback to policy makers through loop R3, enabling them to decide on further investments whenever PR–RP fertilizer price difference increase.

All stakeholders in Budapest and Stockholm believe that increasing initial price of WW treatment in order to enable P recycling is a delicate issue for policy makers. A Swedish farmer association stakeholder explained it differently: “the P-REX project (EU Commission 2017a) concludes that to go from the sludge treatment [we have] today to new technology where we can recover phosphorus, the cost increase [per person] would be roughly 3% of the cost for handling the sewage water […] so if you increase the cost by 3% in Stockholm, you end up with 12 SEK [app. 1.2 euros/month] and that is not even half a cup of coffee”. Therefore, public support for P recycling can also be increased by a more efficient communication on behalf of policy makers (loop R1 in black). Higher public support would give legitimacy to the government to allocate taxpayer money for P recycling. The national-level policy maker interviewee from Hungary as well as the Hungarian NGOs stressed the role of corruption in the system. They believe corruption erodes public trust in the government and decreases available funds for investment. The literature supports this and states that democratic governance and a fair competition are a deficit in the majority of Hungarian public sector organizations that carry out public procurement (Fazekas and Toth 2016). In Fig. 3, corruption decreases public support for P recycling through breach of trust. It also decreases paid out investment subsidies to P recycling projects through graft.

Another factor impacting public support is scientific consensus on sludge spreading safety, which is currently the cheapest form of P recycling. This was a divergent topic for stakeholders in both locations. Stakeholders from academia and the wastewater sector were overwhelmingly supporting sludge spreading, arguing that there is no proof of contamination. On the other hand, one stakeholder from the Swedish farmer association and all stakeholders from the private sector, food industry and the policy-making sectors were either neutral or against sludge spreading, using the argument of the precautionary principle. The food industry representative in Stockholm stated that voluntary initiatives in Sweden such as REVAQ have not yet convinced the industry that sludge spreading is safe. One stakeholder from the Swedish farmer association agreed, stressing that REVAQ only guarantees safe levels for some, but not all heavy metals and pollutants. The Federation of Swedish Farmers now considers the option of withdrawing from REVAQ in 2018 and voted in 2017 to recommend to its farmers not to spread sludge on their land (Land Lantbruk 2017). In 1999, the farmers association recommended that their members stop spreading sludge due to safety concerns (Naturvårdsverket 2011). LRF’s decision at the time, combined with anti-sludge spreading on farmland campaigns from consumer associations has had the effect of most food companies opposing sludge spreading in their supply chain (Bengtsson and Tillman 2004). Moreover, the Swedish Government has recently launched an inquiry into proposing a ban on sewage sludge spreading on agricultural land in July 2018 (Regeringskansliet 2018). One private sector stakeholder from Budapest summarized the safety dilemma with the following: “it's a question of who will take technical, juridical and political responsibility if contamination takes places”. With a high sensitivity to consumer concern, neither the food industry nor the policy makers are willing to take such responsibility. In Sweden, the achievement of phosphorus recycling targets has up until now been reliant on sludge spreading. Latest statistics for 2016 placed the number at 34%, closer to the proposed 2018 target of 40% (SCB 2018). If sludge spreading is banned, achieving the new P recycling targets now implies a rapid optimization of the wastewater system towards P recycling technologies deemed safe. Higher scientific and stakeholder consensus on the safety of recycled P would decrease consumer concern and thus allow the food industry to loosen regulations on the use of recycled P in its supply chain. By doing so, the food industry would positively impact agricultural demand for recycled P and influence the dynamics in loop R2 in Fig. 3. It also gives legitimacy to lawmakers to implement P recycling strategies.

**Fig. 4:**
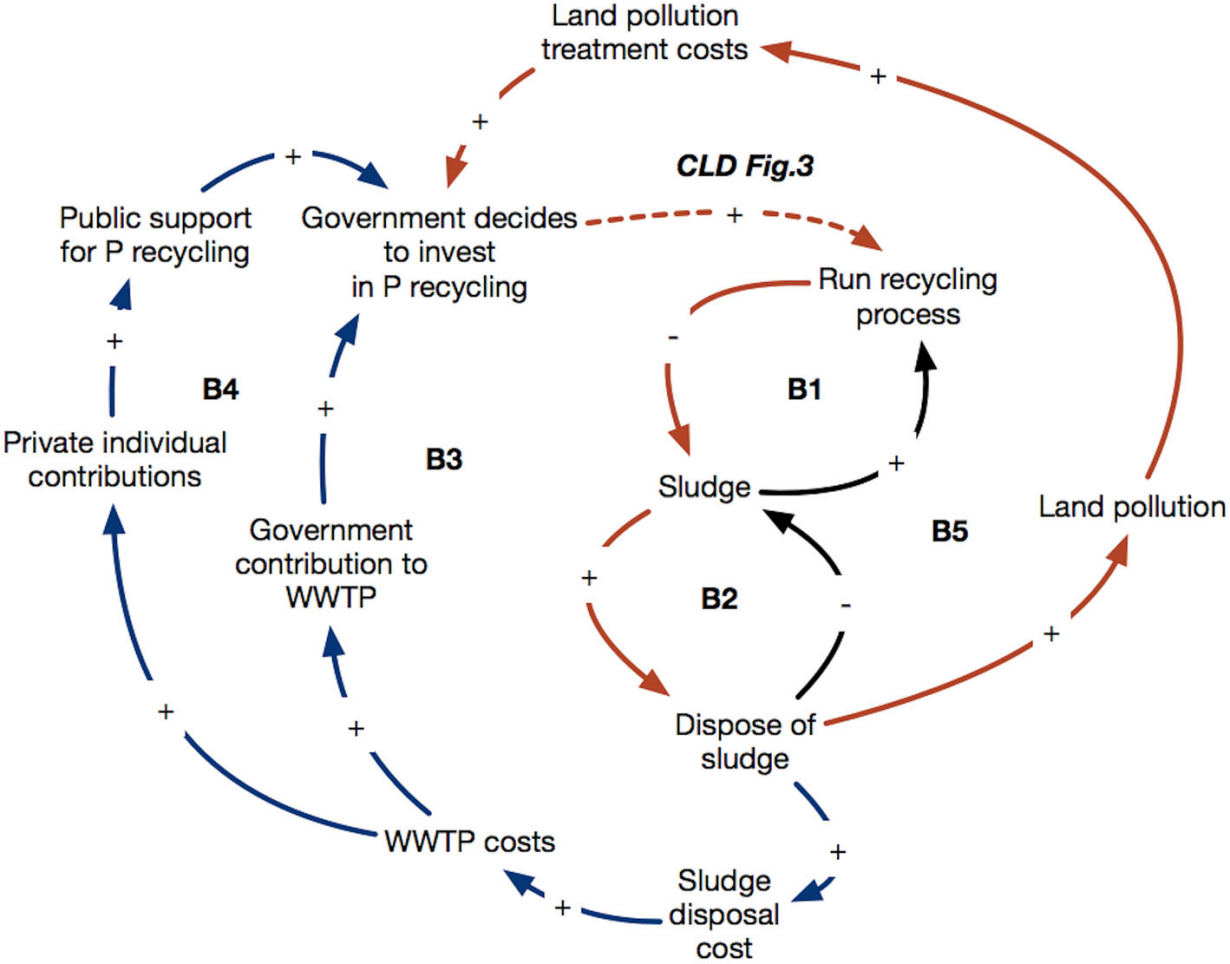
Causal loop diagram on domestic externalities of sludge disposal. Blue arrows are for loops B3 and B4, which show the impact of sludge costs on public support and political support via investment in P recycling. The red arrow is for loop B5, which shows the impact of land pollution of political support via government investment

Molinos-Senante et al. (2011) concluded that P recovery is economically feasible if environmental benefits are considered. The authors looked at 20 WWTPs in Spain and calculated environmental benefits amounting to an average of 42.74 euros for each ton of phosphorus that is not released in the environment. On average, the mean value of environmental benefits was 301 785 euros per WWTP (Molinos-Senante et al. 2011). In Fig. 4 we show the externalities of sludge disposal, with two driving loops: B1 and B2. In B1, P recycling takes out P from sludge and decreases sludge amount. In B2, a lower sludge amount leads to less sludge disposal. Most stakeholders in both Stockholm and Budapest mention that sludge disposal is costly and harmful for the environment. By decreasing the amount of sludge disposal, WWTPs pay less for disposal and lower their overall costs. Lower WWTP costs decrease the price private customers need to pay for wastewater treatment and in turn increases public support for P recycling (loop B4 in blue). As a result, the government also needs to contribute less for wastewater treatment (loop B3 in blue) and pay less for depollution of sludge disposal sites in the future (loop B5 in red).

### Stakeholder analysis

**Fig. 5:**
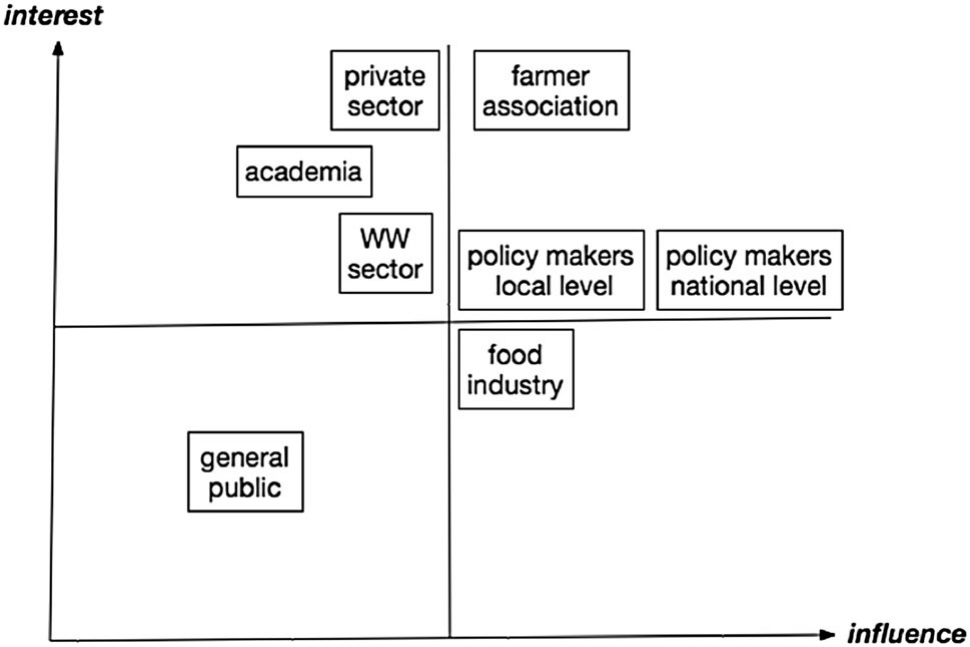
Interest-influence matrix of stakeholders in the P recycling sector

Based on the synthesis presented here, we analyze stakeholders’ role in Stockholm and Budapest in an interest-influence matrix shown in Fig. 5. Policy makers at a national and local level have the highest influence but lack somewhat in interest. This is due to perceived low P criticality, as global fertilizer prices are generally much lower than those of recovered P fertilizers. Also, avoiding conflict between stakeholders is another factor that keeps national and local policy makers reluctant in taking decisive action. Farmers associations have a relatively high influence through their lobby power and a high interest in recycling P.

However, low fertilizer prices and lack of conclusive scientific consensus on recycled P safety prevents them from lobbying more for P recycling. The food industry has influence through its lobby power but less interest due to current low fertilizer prices and concerns about consumers and contamination scandals. To put it in the words of one of the interviewed food industry stakeholders “low P prices means safety wins over recycling at the moment”. Stakeholders in the academic and the private sector have a high interest in the topic but not enough influence—this is due to the perceived lack of urgency for P recycling by stakeholders with higher influence and the small scale of P recycling companies. The interest of wastewater sector stakeholders in Stockholm and Budapest is not that high because binding regulations to recycle P do not exist. General public has in most cases low interest and low influence, unless in exceptional circumstances, such as contamination cases or widespread awareness raising campaigns.

## Discussion

Political support by implementing legally binding P recycling targets is key to developing the P recycling sector. Policy makers are also the most influential stakeholders. Their support for P recycling increases as P criticality increases, which in our analysis has two components: national import dependency and price of imported PR fertilizer (see Fig. 2). Both stakeholders and the literature overwhelmingly point at the economics of P recycling as a sector challenge: the claim is that recycled P price is too high to make recycling profitable.

**Fig. 6:**
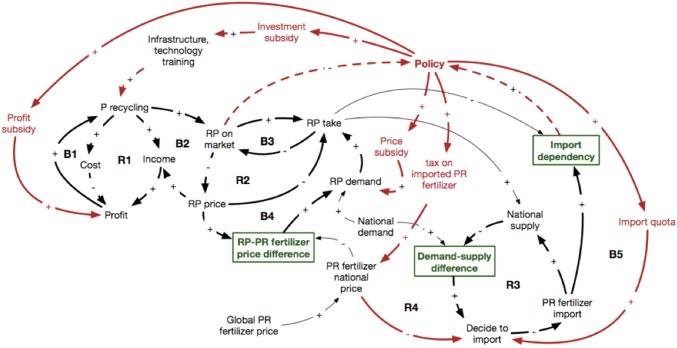
Causal loop diagram showing policy interventions in the P recycling sector (red), their feedback (dashed red) and policy action indicators (green boxes)

We argue that policy makers need to consider more than recycled P fertilizer price. In Fig. 6 we show three indicators for policy action in green bold boxes. First, it is the difference between the price of phosphate rock (PR fertilizer price) and the price of recycled P (RP fertilizer price), which was also presented in Fig. 2. A small or negative difference is an indicator for a profitable P recycling sector. Second, it is the difference between demand and supply. A small or negative difference is a red flag for suboptimal supply of P to the national agricultural sector. Third, it is import dependency, which was presented in Figs. 2 and 3.

In terms of policy interventions, most suggestions from both stakeholders and the literature favor government-lead investment in the wastewater sector. The aim of policy interventions as it emerges from the literature and interviews, is to lower the RP-PR fertilizer price differences. We thus propose four additional entry points in the market system where the state can intervene by setting policies (Fig. 6 in red). First, profit subsidies can intervene in loops R1, B1, and B2 and increase RP on the market by keeping the P recycling sector profitable. Second, price subsidies can increase RP demand by decreasing PR-RP fertilizer price difference (loops R2 and B4). The difference can further be lowered by a third intervention, namely tax on imported PR fertilizer. Lastly, the amount of imported PR fertilizer can be regulated through import quotas. Import quotas will also regulate the share of RP on the market out of the total P fertilizer.

## Conclusion

P is a limited natural resource and an essential macronutrient in agriculture. Its finite resource availability, combined with global supply insecurity, makes it a critical mineral in Europe. Literature shows that recycling P from urban wastewater can secure 20% of the domestic P supply in Europe. Currently, P recycling from urban wastewater is undertaken on a voluntary and small-scale basis in some parts of Europe. Our stakeholder analysis indicates that legally binding targets to recycle P, similar to those in Germany and Switzerland, are needed to improve performance and scale. Previous research showed that such targets can be affordable if P criticality is high. In determining P criticality, we suggest a shadow P fertilizer price to be calculated, taking into account externalities from mine to sludge disposal. According to both the literature and our stakeholder analysis, national import dependency is another key aspect of criticality that policy makers should account for. Policy interventions need to account for high initial costs to establish recycling and the delay in the system to reach recycling profitability. P recycling strategies need to sufficiently address public concerns on health and safety, costs and the reasoning behind P recycling. Past experience in Sweden showed that the decades long sewage sludge debate caused significant delay in implementing a P recycling strategy. Our interviews suggest that scientific and stakeholder consensus on P recycling safety is required when designing such a strategy. Improving P recycling in Europe will support the circular economy EU regulation from 2015 and expand the circular economy action plan of 2018.

## Electronic supplementary material

Below is the link to the electronic supplementary material.

Supplementary material 1 (PDF 91 kb)
