# Supplementary material for: From waste to resource: A systems dynamics and stakeholder analysis of phosphorus recycling from municipal wastewater in Europe
Source: Ambio. 2018 Sep 14;48(7):741–51. doi: 10.1007/s13280-018-1097-9 (PMC6509087; doi:10.1007/s13280-018-1097-9)
Supplement: Supplementary file 1 — Supplementary material 1 (PDF 91 kb) [file 13280_2018_1097_MOESM1_ESM.pdf]

Ambio
https://doi.org/10.1007/s13280-018-1097-9

Electronic Supplementary Material

From waste to resource: A systems dynamics and stakeholder analysis of phosphorus recycling from municipal wastewater in Europe

Claudiu-Eduard Nedelciu, Kristín Vala Ragnasdóttir, Ingrid Stjernquist

Annex S1

Guiding questions interviewees were asked in Sweden and Hungary.

1. Do you think it is important to recover Phosphorus (P) in urban areas? Why?
2. How economically viable is it to recover P and which is the most cost-effective yet safest wastewater treatment (WWT) technology that can be applied? Are there any ways to further reduce costs?
3. In your opinion, which are the main challenges in recycled P from urban wastewater?
4. Which are the social obstacles in reusing recovered P and which do you think are the solutions in solving them?
5. Do you think pharmaceuticals in wastewater are an issue for P recovery or is the WWT at the moment sufficient to keep those at a safe level?
6. What could policy makers do better and at which level? (municipal/national/EU)
7. When do you see Sweden/Hungary achieving its objectives on P recovery?
